# Structural, optical and photocatalytic properties of flower-like ZnO nanostructures prepared by a facile wet chemical method

**DOI:** 10.3762/bjnano.4.87

**Published:** 2013-11-18

**Authors:** Sini Kuriakose, Neha Bhardwaj, Jaspal Singh, Biswarup Satpati, Satyabrata Mohapatra

**Affiliations:** 1 School of Basic and Applied Sciences, Guru Gobind Singh Indraprastha University, Dwarka, New Delhi 110078, India; 2 Saha Institute of Nuclear Physics, 1/AF Bidhannagar, Kolkata 700064, India

**Keywords:** ageing, nanoparticles, nanosheets, photocatalysis, ZnO

## Abstract

Flower-like ZnO nanostructures were synthesized by a facile wet chemical method. Structural, optical and photocatalytic properties of these nanostructures have been studied by X-ray diffraction (XRD), scanning electron microscopy (SEM), transmission electron microscopy (TEM), photoluminescence (PL) and UV–vis absorption spectroscopy. SEM and TEM studies revealed flower-like structures consisting of nanosheets, formed due to oriented attachment of ZnO nanoparticles. Flower-like ZnO structures showed enhanced photocatalytic activity towards sun-light driven photodegradation of methylene blue dye (MB) as compared to ZnO nanoparticles. XRD, UV–vis absorption, PL, FTIR and TEM studies revealed the formation of Zn(OH)_2_ surface layer on ZnO nanostructures upon ageing. We demonstrate that the formation of a passivating Zn(OH)_2_ surface layer on the ZnO nanostructures upon ageing deteriorates their efficiency to photocatalytically degrade of MB.

## Introduction

Water contamination due to hazardous water soluble organic dyes and chemicals poses a severe threat to the environment. The excess azo dyes in effluents from textile and dyeing industries are usually resistant to biodegradation. Due to their stability and large degree of organics present in them, these pollutants pose severe ecological problems by depleting the dissolved oxygen content of water and releasing toxic compounds that endanger the aquatic life. During an anaerobic treatment, these azo dyes may generate carcinogenic compounds such as aromatic amines. Because of this, purification and detoxification of industrial waste water has been one of the major challenges. Several methods such as adsorption, filtration, sedimentation and photocatalysis are used for the removal of these toxic chemicals. Photocatalytic degradation, in which the organic pollutants are degraded through photocatalytic oxidation and reduction reactions in the presence of a photocatalyst, is one of the most promising and clean processes used for water purification.

Nanostructured semiconductor photocatalysts such as ZnO and TiO_2_ have attracted significant attention in recent years because of their wide-spread application in environmental remediation [1–2]. These photocatalysts have a high efficiency for the degradation of toxic organic pollutants that originate from the effluents of textile and dyeing industries. Since the sun is an abundantly available natural energy source, its light can be conveniently utilized for the photodegradation of organic dyes [3–8]. ZnO with a band gap of 3.37 eV has received much attention for the complete mineralization and degradation of environmental pollutants. ZnO nanostructures with different morphologies have been synthesized by wet chemical methods [9–13] and used for various applications such as photocatalytic degradation of organic dyes [14–24], dye sensitized solar cells [25–28], gas sensors [29–30], clean energy applications [31] and UV detection [32]. Xia et al. [12] synthesized nanostructured ZnO flowers made up of bundled nanochains that could detect dopamine in the presence of L-ascorbic acid with high sensitivity and selectivity. Flower-shaped ZnO nanostructures were synthesized by Umar et al. [31] for an efficient photocatalysis and the fabrication of efficient dye sensitized solar cells. Shi et al. [33] fabricated flower-like ZnO on ZnO nanorods without use of any surfactant. Self-supported ZnO photocatalysts in the form of plates were prepared by Yassitepe et al. [24] by the tape casting method. These ZnO plates showed a good photocatalytic activity for azo dyes that depended on their surface area. Shen et al. [34] have shown that depositing ZnO on silica nanoparticles is a simple and effective method to prepare photocatalysts that could degrade 90% methylene blue (MB) in 60 min. ZnO nanoparticles (NP) that were synthesized by wet chemical methods can be passivated by a Zn(OH)_2_ layer during ageing. Hong et al. [35] have shown that the photocatalytic activity of ZnO NP is drastically reduced when the is surface modified with polysterene. In this paper, we have studied the effects of ageing on structural, optical and photocatalytic properties of flower-like ZnO nanostructures, which were prepared by a simple wet chemical method. We have demonstrated that the formation of a passivating layer of Zn(OH)_2_ on the ZnO nanostructures due to ageing leads to a drastic decrease in the efficiency of the sunlight driven photocatalytic degradation of MB.

## Results and Discussion

**Figure 1:**
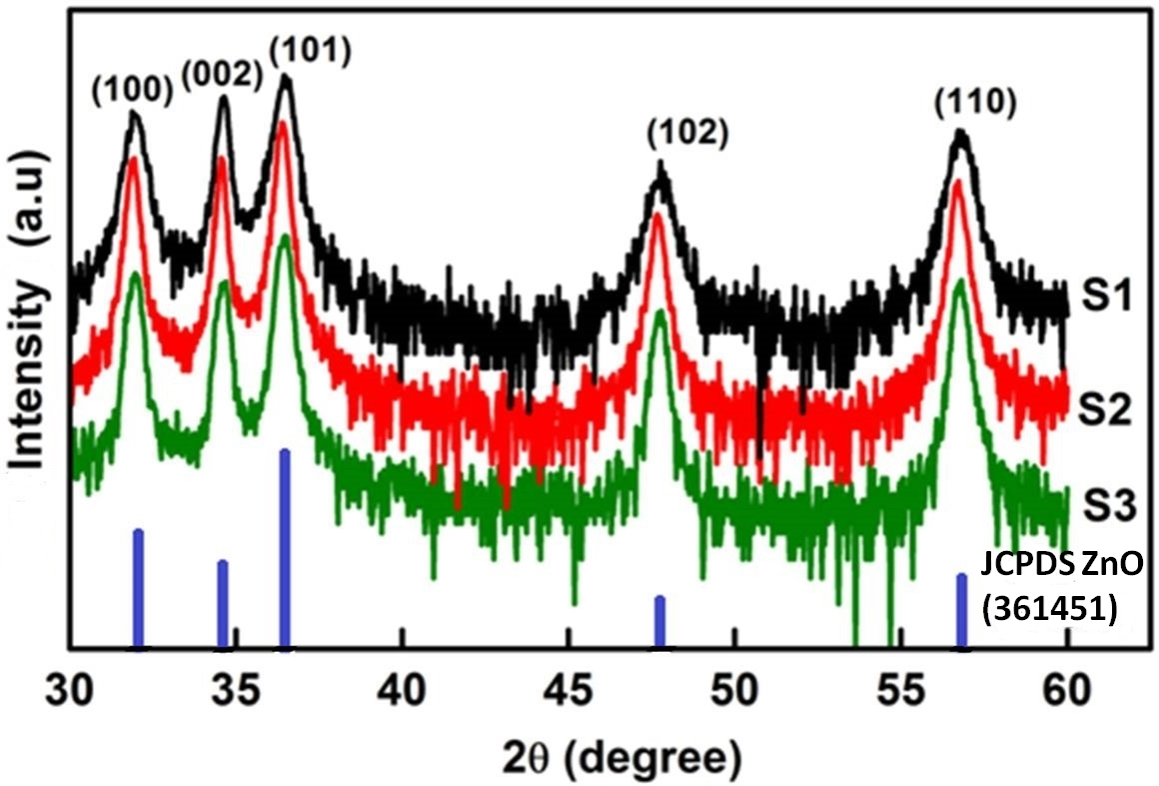
XRD patterns of samples S1, S2 and S3 in log scale indexed to the hexagonal wurtzite structure of ZnO. The standard JCPDS data for ZnO is also shown for comparision.

Figure 1 shows the XRD patterns of the as-synthesized samples S1, S2 and S3 (see Experimental section for the naming scheme). The observed well-defined peaks in the spectra can be indexed to the hexagonal wurtzite structure of bulk crystalline ZnO [JCPDS no. 36-1451]. No extra peaks related to any impurity were observed. This confirms that the as-synthesized products are pure wurtzite-type ZnO.

**Figure 2:**
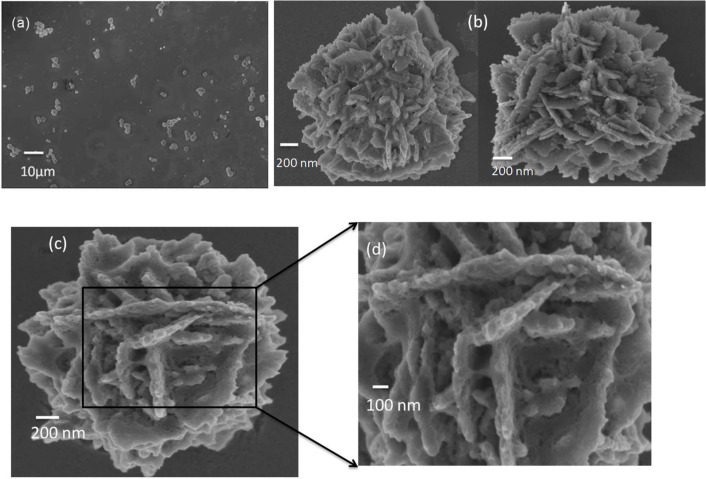
SEM image of sample S2 showing flower-like structures consisting of nanosheets made up of nanoparticles.

Figure 2a shows a low magnification FESEM image of sample S2, which reveals flower-like structures with different forms throughout the sample. The higher magnification images of these structures are shown in Figure 2b and Figure 2c. Figure 2c shows a FESEM image of one such structure and its magnified image is shown in Figure 2d. It can be clearly seen that these flower-like structures are made up of nanosheets. These nanosheets were found to consist of nanoparticles and display a porous morphology. In addition to these flower-like structures, a large number of nanoparticles can also be seen.

### Mechanism of formation of flower-like ZnO structures

**Figure 3:**
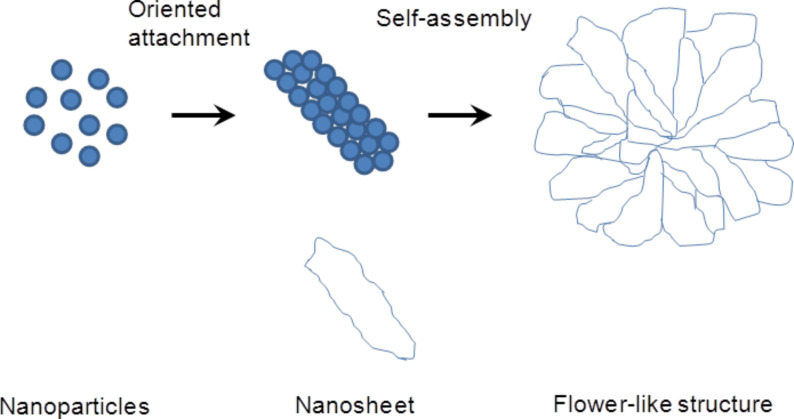
Schematic diagram depicting mechanism for formation of flower-like ZnO structures involving (i) formation of ZnO spherical nanoparticles, (ii) oriented attachment of ZnO nanoparticles resulting in nanosheets, followed by (iii) self-assembly of nanosheets into flower-like ZnO structures.

On the basis of the FESEM results and earlier works on the synthesis of ZnO flowers [36–38], it is proposed that the formation of flower-like ZnO structures involves three stages, which are schematically depicted in Figure 3. The growth of flower-like ZnO structures strongly depends on the concentration of Zn^2+^ and OH^−^ ions and involves the following processes: (i) nucleation and growth of ZnO nanoparticles, (ii) oriented attachment of ZnO nanoparticles to form nanosheets and (iii) self-assembly of nanosheets into three-dimensional flower-like structures. In the present study, ZnO nanostructures have been synthesized by a facile wet chemical method, which uses zinc acetate and KOH as precursors mixed in a ratio of 1:10 under stirring at 60 °C. It is well known that the formation of ZnO nanoparticles in aqueous solutions from Zn(CH_3_COO)_2_ and KOH under alkaline conditions and heating involves the following reactions [39–40]:

[1]
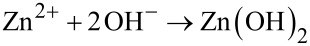


[2]
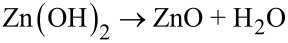


[3]
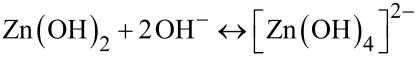


[4]
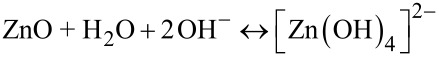


Firstly, the addition of KOH into an aqueous solution of Zn(CH_3_COO)_2_ leads to the formation of white precipitates of Zn(OH)_2_ (Equation 1), which upon heating decompose to form ZnO nuclei (Equation 2). Depending on the Zn^2+^ concentration and synthesis conditions, the ZnO nuclei grow into nanoclusters. In the presence of excess OH^−^ ions (in our case a much higher KOH concentration), the formation of [Zn(OH)_4_]^2−^ ions is preferred (Equation 3). Dehydration of [Zn(OH)_4_]^2−^ due to heating leads to nucleation and growth of ZnO nanoparticles (Equation 4). The [Zn(OH)_4_]^2−^ complexes preferentially adsorb onto the surface of the ZnO nanoparticles and facilitate their easy growth along the c-axis to minimize the energy of the hexagonal crystal structure [40]. This leads to an oriented colaescence of ZnO nanoparticles resulting in the formation of ZnO nanosheets. It is interesting to note that the nanosheets have some degree of porosity (Figure 2d). Defects on the ZnO nanosheets act as nucleation sites for the growth of secondary nanosheets. The primary and secondary nanosheets self-assemble to minimize the surface energy, and this leads to the formation of three-dimensional flower-like ZnO structures [30,40].

### Photocatalytic studies

**Figure 4:**
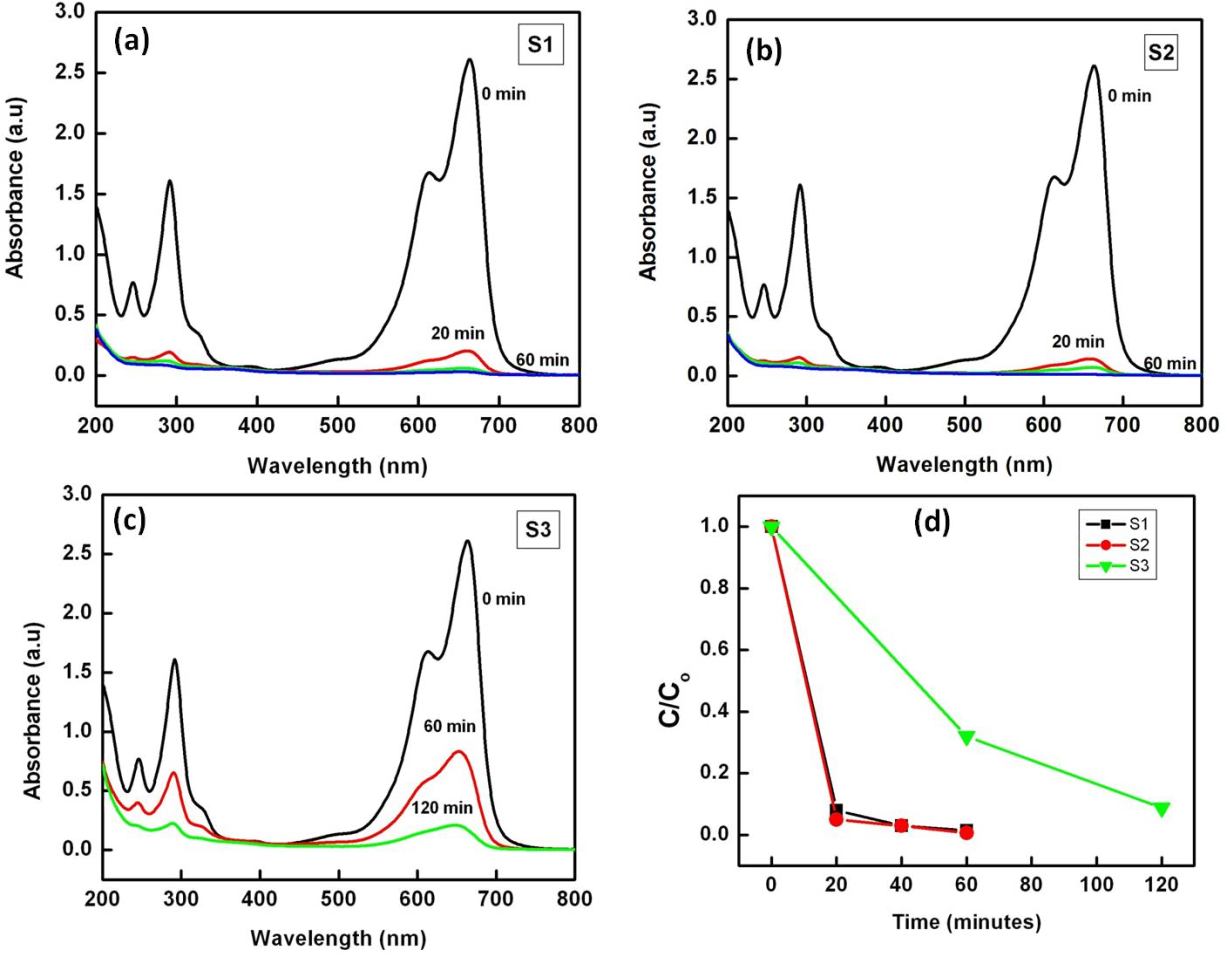
UV–vis absorption spectra showing temporal evolution of photocatalytic degradation of MB upon irradiation with sun light using samples (a) S1, (b) S2 and (c) S3 as photocatalysts. (d) variation of MB concentration with exposure time for different photocatalysts.

Figures 4a–c show the UV–vis absorption spectra of an aqueous solution of 22.4 μM MB with either photocatalyst S1, S2 or S3 after irradiation with sunlight for different durations of time. The characteristic absorption peak of MB at 664 nm is monitored as a function of the sunlight exposure time. It can be seen that the absorption peak at 664 nm diminishes sharply after 20 min of irradiation and almost completely disappears after 60 min of irradiation with the photocatalysts S1 and S2. However, the absorption peak for the sample with S3 shows a much slower reduction in intensity than S1 and S2 and is still clearly visible even after 120 min of irradiation. No new absorption peaks appear in the UV–vis region, which clearly indicates the complete photocatalytic degradation of MB. It must be pointed out here that an adsorption of MB onto the ZnO nanostructures in dark prior to sunlight exposure did not result in any significant change in the absorption spectrum of MB. Figure 4d shows the extent of photodegradation of MB marked by the changes in *C*/*C*_0_ with irradiation time. Irradiation with sunlight for 20 min resulted in 92% degradation of MB when sample S1 was used, whereas by using sample S2 we could achieve about 95% degradation of MB for the same exposure time. Almost complete (99.6%) photodegradation of MB could be achieved with S2 as photocatalyst after 60 min of exposure to sunlight, while samples S1 and S3 could degrade only 97.8% and 68.2% respectively after the same exposure time.

The efficiency of the photocatalysts after 60 min of sunlight exposure is given in Table 1. Sample S2 was found to have the highest photocatalytic degradation efficiency (99.6%). It is important to mention here that photocatalytic studies under direct sunlight irradiation are better for use as compared to the use of high-pressure mercury lamp and Xe lamp as light sources.

**Table 1:** Photodegradation efficiency of different photocatalysts used.

sample	irradiation time (min)	photodegradation efficiency, η (%)

S1	60	97.8
S2	60	99.6
S3	60	68.2

**Figure 5:**
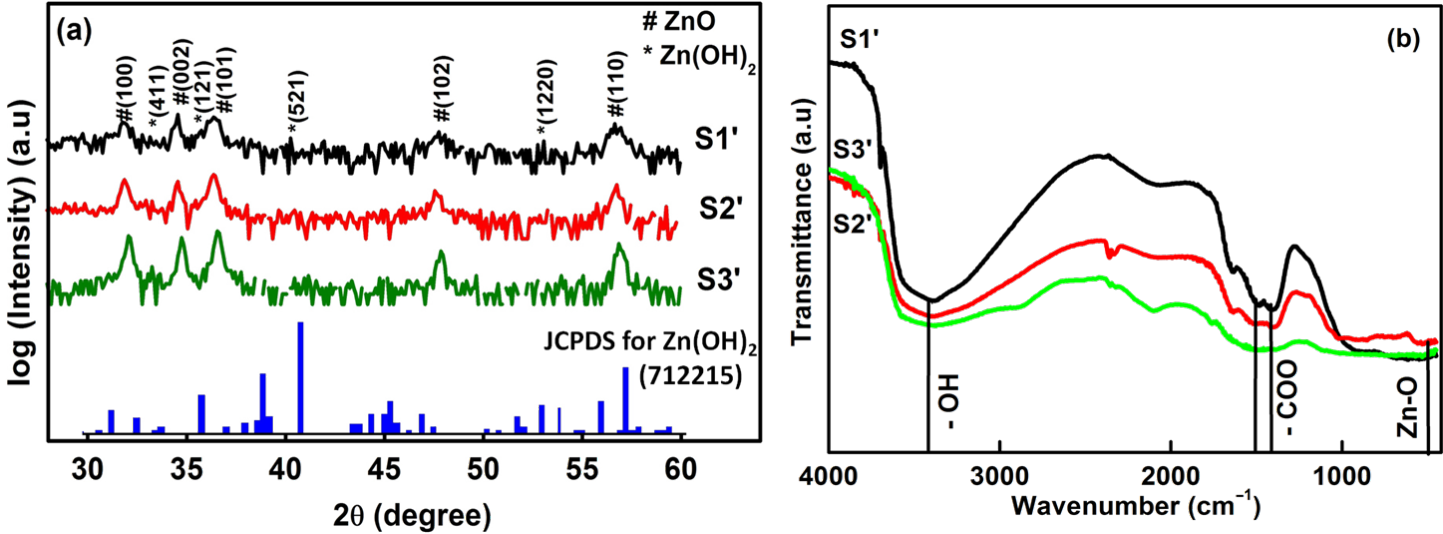
(a) XRD patterns of aged samples S1’, S2’ and S3’ in log scale indexed with peaks of ZnO and Zn(OH)_2_, (b) FTIR spectra of the aged samples S1’, S2’ and S3’ showing various groups present in the sample.

To check the effects of ageing on the photocatalytic efficiency, the as-synthesized ZnO samples were stored in dark and under humid conditions for 12 months. After that, the photocatalytic experiment was repeated again with sample S2’ (see section Experimental for the naming scheme), because it had the maximum photodegradation efficiency before ageing. Figure 5a shows the XRD patterns of the aged samples S1’, S2’ and S3’, which along with the peaks of hexagonal wurtzite ZnO also show additional small peaks corresponding to Zn(OH)_2_. The presence of Zn(OH)_2_ is also supported by the FTIR data shown in Figure 5b. The peak at 472 cm^−1^ is the characteristic absorption of the Zn–O bond [35]. Features appearing between 1400 and 1600 cm^−1^ that comprise of several peaks can be attributed to the stretching modes (symmetric and asymmetric) of the acetate group (–COO) [41] that may have been adsorbed during the synthesis process. The presence of the hydroxy group (–OH) can be confirmed by the presence of a broad feature around 3400 cm^−1^ [42], which corresponds to the stretching vibration of the –OH bond.

**Figure 6:**
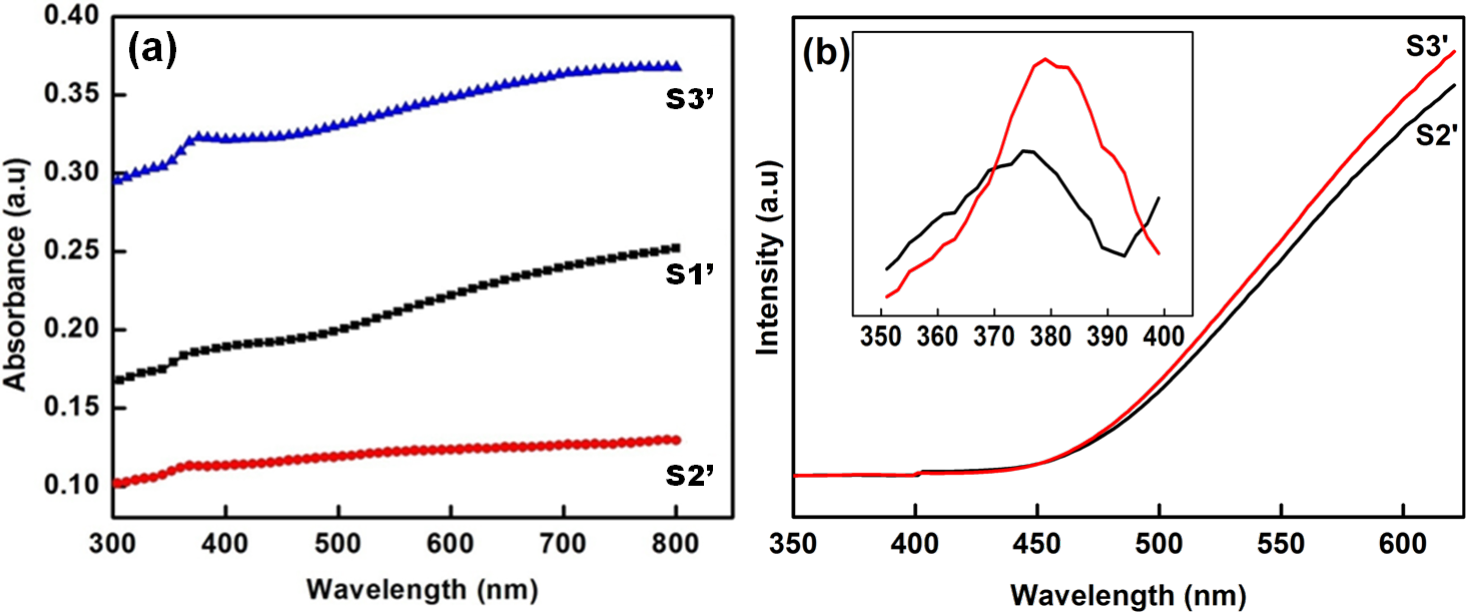
(a) UV–vis absorption spectra of aged samples S1’, S2’ and S3’, (b) room temperature PL spectra, inset showing the NBE emission peak in the aged ZnO samples.

**Figure 7:**
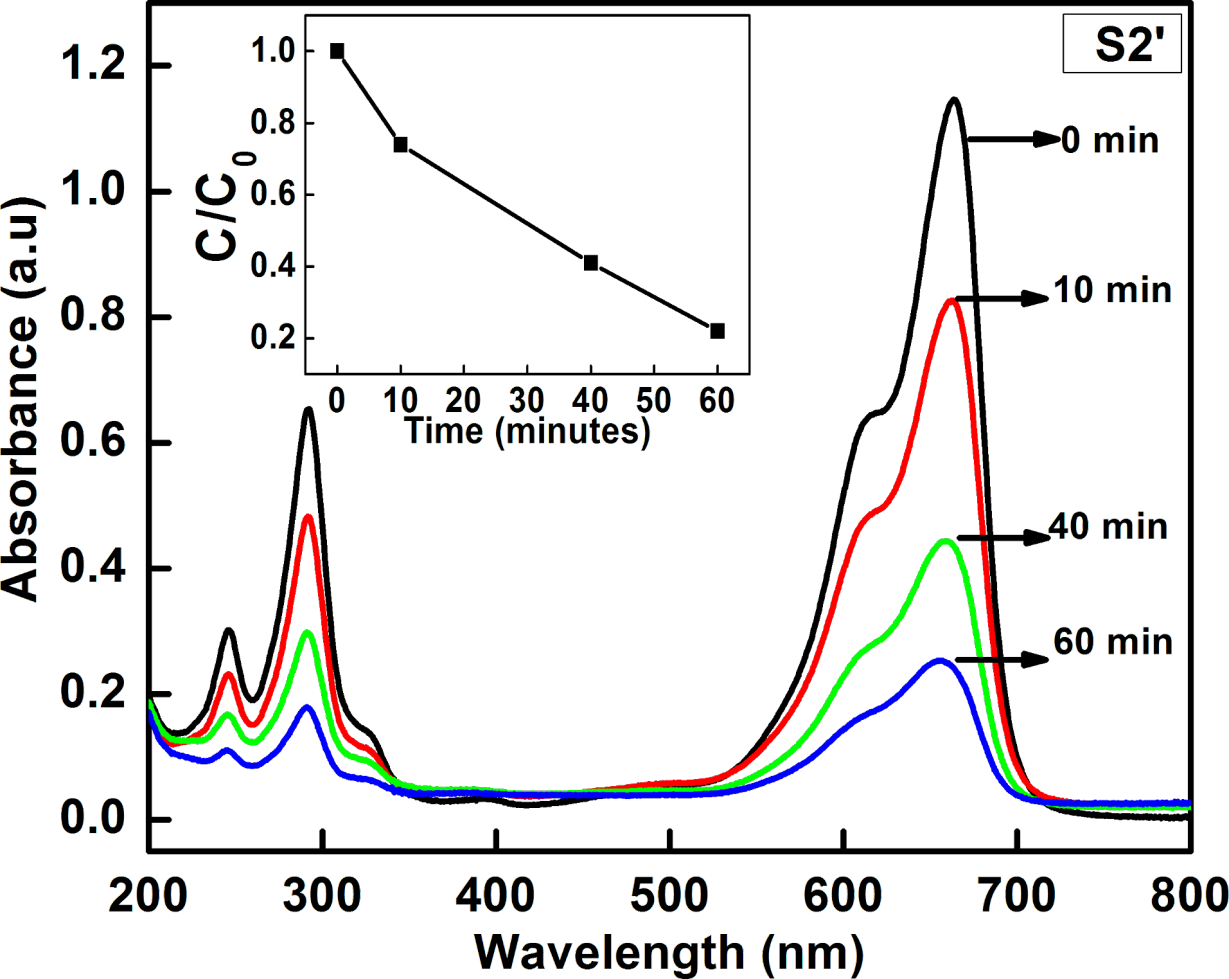
UV–vis absorption spectra showing photocatalytic degradation of 10 μM MB upon irradiation with sun light using aged sample S2’ as photocatalyst. Inset shows the variation of MB concentration with exposure time.

UV–vis absorption spectra of S1’, S2’ and S3’ are shown in Figure 6a. A small peak can be seen in the UV region and a broad band in the visible region was observed. The broad band in visible region may be due to the presence of Zn(OH)_2_. The presence of Zn(OH)_2_ passivating layer on ZnO nanostructures can also be seen from the PL spectra. Figure 6b shows the room temperature PL spectra of aged samples S2’ and S3’ showing an enhanced visible light emission and a suppressed near band edge (NBE) emission. The weak NBE emission from the ZnO nanocrystals strongly indicates the presence of a passivating Zn(OH)_2_ surface layer [16], as confirmed by the XRD data. In an earlier work, Zhou et al. [16] have shown that the formation of a Zn(OH)_2_ shell on ZnO nanocrystals leads to a drastic decrease in the NBE emission along with an enhancement in the visible emission. In our case, the observed suppression of NBE emission and enhancement in visible emission from ZnO nanostructures upon ageing is due to their surface modification with Zn(OH)_2_, which results in a decrease of the crystalline quality. Figure 7 shows the UV–vis absorption spectra of a 10 μM MB solution with sample S2’ as the photocatalyst. It can be seen that the photodegradation efficiency of the aged sample S2’ (78.2% of 10 μM MB) is much less than the efficiency of sample S2 (99.6% of 22.4 μM MB) for 60 min of irradiation with sun light. This shows that the formation of a Zn(OH)_2_ surface layer deteriorates the photocatalytic efficiency of ZnO.

The enhanced photocatalytic activity of flower-like ZnO nanostructures as compared to ZnO nanoparticles can be attributed mainly to the large surface area, which arises from the three-dimensional flower-like ZnO structures consisting of porous nanosheets. The large surface area results in an enhanced adsorption of MB. Earlier studies have shown that the surface morphology of the ZnO nanostructures plays a very important role in the photocatalytic activity [17,19–21,43]. Differences mainly originate from differences in surface area, polar planes or oxygen vacancies. The processes underlying the photocatalytic degradation of MB can be understood as follows. The first step involves adsorption of MB onto the ZnO nanostructures. Irradiation with sunlight leads to the generation of electron–hole (e^−^–h^+^) pairs in ZnO (Equation 5). The electrons in the conduction band of ZnO interact with oxygen molecules adsorbed on ZnO to form superoxide anion radicals (•O_2_^−^) (Equation 6). The holes generated in the valence band of ZnO react with surface hydroxy groups to produce highly reactive hydroxyl (•OH) radicals (Equation 7). The photogenerated holes can lead to the production of •OH radicals through the dissociation of water (Equation 8). The highly reactive hydroxyl radicals (•OH) and superoxide radicals (•O_2_^−^) react with MB adsorbed on ZnO nanostructures and lead to the degradation of MB. These underlying processes can be summarized by the equations [44].

[5]
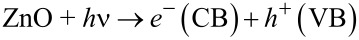


[6]
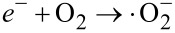


[7]
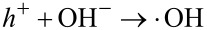


[8]
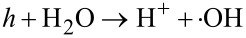


[9]
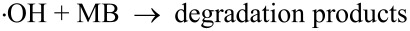


[10]
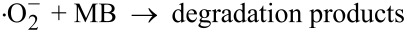


## Conclusion

In summary, we have synthesized flower-like ZnO nanostructures by using a facile wet chemical method. The structural, optical and photocatalytic properties of ZnO nanostructures have been investigated. As compared to spherical ZnO nanoparticles, flower-like ZnO nanostructures exhibited an enhanced photocatalytic efficiency towards the degradation of methylene blue dye under irradiation with sunlight. The effect of ageing of flower-like ZnO structures on their photocatalytic efficiency has been investigated. XRD, FTIR, PL and UV–vis absorption studies confirmed the formation of a Zn(OH)_2_ layer on ZnO nanostructures upon ageing. Photocatalytic studies showed that Zn(OH)_2_ surface passivating layer leads to a drastic reduction in the efficiency of flower-like ZnO structures for sunlight induced photocatalytic degradation of MB dye.

## Experimental

### Materials

Zinc acetate dihydrate (Zn(CH_3_COO)_2_·2H_2_O) and potassium hydroxide (KOH) were used as the starting materials for synthesis of ZnO nanostructures. Zinc acetate dihydrate, KOH and methylene blue (MB) were purchased from SRL, India. All chemicals were of analytical grade and used as received without any further purification.

### Synthesis of ZnO nanostructures

ZnO nanostructures were synthesized by a simple wet chemical route using alkali precipitation from aqueous solutions of Zn(CH_3_COO)_2_·2H_2_O and KOH. In a typical synthesis, calculated amounts of Zn(CH_3_COO)_2_·2H_2_O was added into 100 mL of doubly distilled water under stirring to prepare 0.02 M, 0.05 M and 0.1 M solutions. Different amounts of KOH were added directly into these solutions at 60 °C under stirring to reach concentrations 10 times higher than the corresponding Zn^2+^ concentration. The stirring was continued for 3 h with the temperature being maintained at 60 °C. Subsequently, the solution was allowed to cool down and left undisturbed overnight. The white precipitates formed were centrifuged and thoroughly washed by repeated centrifugation–redispersion cycles with doubly distilled water. After this, the precipitates were dried overnight in an oven at 80 °C and the obtained solid white powder was used for characterization and photocatalytic studies. The samples prepared by using different zinc acetate concentrations of 0.02 M, 0.05 M and 0.1 M are referred to as S1, S2 and S3.

### Characterization of photocatalysts

The structural properties of the samples were determined by powder X-ray diffraction (XRD) at room temperature using Panalytical X’pert Pro diffractometer with Cu Kα radiation (λ = 0.1542 nm). Scanning electron microscopy (SEM) was also used for studying the morphology of ZnO nanostructures. Transmission electron microscopy (TEM) investigations were carried out using an FEI Tecnai G^2^ F30 S-Twin microscope operating at 300 kV. FTIR spectra of the samples were recorded in the range from 400 to 4000 cm^−1^. The optical properties of the samples were studied by UV–vis absorption spectroscopy and photoluminescence (PL) spectroscopy at room temperature. The powder samples were dispersed in doubly distilled water by sonication and their optical properties were studied by UV–vis absorption spectroscopy in the wavelength range from 200 to 800 nm using a HITACHI U3900 spectrophotometer with doubly distilled water as the reference medium.

### Photocatalytic measurements

The photocatalytic performance of the ZnO nanostructures were evaluated by measuring the degradation of MB under sunlight irradiation. Aqueous solutions of 22.4 μM MB were prepared in 100 mL doubly distilled water. For the photocatalytic studies, typically 5 mg of the as-synthesized ZnO nanostructures as photocatalysts were ultrasonically dispersed the MB solutions in 10 mL glass vials, which were used as the reactors. The MB solutions with ZnO photocatalysts were thoroughly mixed and kept in the dark for 30 min to reach the adsorption−desorption equilibrium. The reaction suspensions containing MB and nanostructured ZnO photocatalysts were irradiated with sunlight for different times (10, 20, 40, 60, 120 min) under intermittent shaking for uniform mixing of the photocatalysts with the MB solution. These experiments were carried out ensuring irradiations with sunlight of maximum luminosity. Then the suspensions were centrifuged and the photocatalysts were removed from the suspension. The concentrations of MB in the resultant solutions were monitored by UV–vis absorption spectroscopy in the wavelength range from 200 to 800 nm, with doubly distilled water as the reference medium. The photocatalytic degradation efficiency for MB was calculated by using the following formula:

[11]
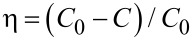


where *C*_0_ is the absorbance of the aqueous MB solution before addition of the photocatalyst and exposure and *C* is the absorbance of MB in reaction suspensions with photocatalyst after exposure to sunlight for the time *t*.

In order to study the effect of ageing of ZnO nanostructures on the sunlight driven photocatalytic degradation of MB, the as-synthesized samples (S1, S2 and S3) were stored in dark and under humid conditions for 12 months and photocatalytic experiments were repeated again on the aged ZnO samples (referred to as S1’, S2’ and S3’).
